# Patent and Bibliometric Analysis of the Scientific Landscape of the Use of Pulse Oximeters and Their Prospects in the Field of Digital Medicine

**DOI:** 10.3390/healthcare11223003

**Published:** 2023-11-20

**Authors:** Olena Litvinova, Fabian Peter Hammerle, Jivko Stoyanov, Natalia Ksepka, Maima Matin, Michał Ławiński, Atanas G. Atanasov, Harald Willschke

**Affiliations:** 1 Department of Management and Quality Assurance in Pharmacy, National University of Pharmacy, Ministry of Health of Ukraine, 61002 Kharkiv, Ukraine; 2 Ludwig Boltzmann Institute Digital Health and Patient Safety, Medical University of Vienna, 1090 Vienna, Austria; fabian.hammerle@meduniwien.ac.at; 3 Department of Anesthesia, General Intensiv Care and Pain Management, Medical University of Vienna, 1090 Vienna, Austria; 4 Swiss Paraplegic Research, 6207 Nottwil, Switzerland; jivko.stoyanov@paraplegie.ch; 5 Institute of Genetics and Animal Biotechnology of the Polish Academy of Sciences, 05-552 Magdalenka, Poland; n.ksepka@igbzpan.pl (N.K.); m.matin@igbzpan.pl (M.M.); m.lawinski@igbzpan.pl (M.Ł.); 6 Department of General, Gastroenterologic and Oncologic Surgery, Medical University of Warsaw, 02-097 Warsaw, Poland

**Keywords:** pulse oximeters, patient safety, digital medicine, artificial intelligence, machine learning, telemedicine, patent

## Abstract

This study conducted a comprehensive patent and bibliometric analysis to elucidate the evolving scientific landscape surrounding the development and application of pulse oximeters, including in the field of digital medicine. Utilizing data from the Lens database for the period of 2000–2023, we identified the United States, China, the Republic of Korea, Japan, Canada, Australia, Taiwan, and the United Kingdom as the predominant countries in patent issuance for pulse oximeter technology. Our bibliometric analysis revealed a consistent temporal trend in both the volume of publications and citations, underscoring the growing importance of pulse oximeters in digitally-enabled medical practice. Using the VOSviewer software(version 1.6.18), we discerned six primary research clusters: (1) measurement accuracy; (2) integration with the Internet of Things; (3) applicability across diverse pathologies; (4) telemedicine and mobile applications; (5) artificial intelligence and deep learning; and (6) utilization in anesthesiology, resuscitation, and intensive care departments. The findings of this study indicate the prospects for leveraging digital technologies in the use of pulse oximetry in various fields of medicine, with implications for advancing the understanding, diagnosis, prevention, and treatment of cardio-respiratory pathologies. The conducted patent and bibliometric analysis allowed the identification of technical solutions to reduce the risks associated with pulse oximetry: improving precision and validity, technically improved clinical diagnostic use, and the use of machine learning.

## 1. Introduction

Patient safety remains a paramount concern across multiple fields of medicine, including anesthesiology, intensive care, resuscitation, surgery, and pediatrics. A critical diagnostic and prognostic factor for ensuring patient safety is the continuous monitoring of blood oxygen saturation levels. This metric serves as a comprehensive indicator of the respiratory and cardiovascular system functioning. Pulse oximetry has emerged as a non-invasive, safe, and highly informative method for monitoring the percentage of oxyhemoglobin in arterial blood [1].

Blood oxygen saturation monitoring is widely used in many areas of medicine, not only for patients but also for healthy individuals, to assess the cardiorespiratory status [2]. Pulse oximetry is a standard of mandatory monitoring protocols during anesthesia, as it detects episodes of hypoxia and allows timely medical intervention. During the awakening period and throughout patients’ stay at the post-anesthesia care unit, pulse oximetry serves as a standard component of monitoring the restoration of vital functions, particularly adequate breathing and blood circulation [3]. Pulse oximetry does not only provide oxygen saturation levels, but also provides pulse frequency, pulse regularity, and respiratory variation in the amplitude of the plethysmographic waveform (as a predictor of fluid responsiveness), thus helping anesthesiologists decide whether a patient would benefit from intervening fluids.

It is worth noting that pulse oximetry is used in any setting where a patient’s oxygenation may be unstable. This includes intensive care units, surgical theaters, recovery rooms, emergency departments, and general hospital wards. It is used to assess any patient’s oxygenation, determine the need for additional oxygen, and detect life-threatening conditions in newborns and premature infants [4,5,6,7]. Additionally, pulse oximeters find utility in healthy people in the training and testing of pilots, firefighters, military personnel, professional athletes, and mountain climbers [8,9,10,11].

One of the biggest achievements in the history of clinical monitoring was the pulse oximeter [12,13]. For more than 30 years of application, pulse oximetry has not lost its relevance. The World Federation of Societies of Anesthesiologists, the American Society of Anesthesiologists, and the World Health Organization advise the use of pulse oximetry as part of standard intraoperative monitoring [14,15].

Marketing analysis data indicate that the market for pulse oximeters was valued at USD 2.1 billion in 2021 and is anticipated to increase to USD 3.9 billion by 2031, with a compound annual growth rate (CAGP) of 6.3% from 2022 to 2031 [16]. This market expansion is attributed to the rise of cardiovascular and respiratory diseases, diabetes, and aging populations. In addition, the increase in surgical procedures and hospitalizations, combined with technological advances in pulse oximeters, are additional factors driving market demand. The COVID-19 pandemic has further accelerated the adoption of pulse oximeters, both in clinical settings and for home use. In patients with respiratory conditions, oxygen saturation serves as a key metric for assessing the effectiveness of pulmonary gas exchange efficiency and treatment efficacy [17,18,19,20,21].

Optical oximetry methods are well-established and promising in the context of modern evidence-based medicine. They have a solid physical and mathematical foundation, and are based on the methods of physical measurements, i.e., they are a full-fledged branch of modern medical physics. Despite their robust foundations, questions remain regarding the accuracy and reproducibility of the results of such measurements related to the effects of pigmentation, race, fingernail polish, ambient light, and motion artifacts [22,23,24,25]. Notably, in November 2022, the US FDA recommended improvements to pulse oximeters following studies that indicated reduced efficacy in individuals with darker skin [26].

The implementation of digital technologies while using pulse oximeters is also promising, including remote monitoring, and artificial intelligence is an area of research interest today in major scientific centers around the world. This diagnostic direction, in the full sense of the word, is an evolving scientific frontier [27,28,29,30].

This study’s objective was to conduct a patent and bibliometric analysis of the scientific landscape of contemporary innovative technological solutions for the development and application of pulse oximeters, including in the field of digital medicine, and identify technical solutions to reduce the risks associated with the use of pulse oximetry.

## 2. Materials and Methods

When investigating this topic, an analysis of patents and scientific information was carried out. General scientific and specific scientific research methods were applied (systematic approach, method of analysis, synthesis, induction and deduction, etc.).

### 2.1. Patent Analysis

Patents are indicators of technological activity and innovative potential. The information presented in the patents is unique and does not always appear in other publications. Patent databases store and constantly update huge amounts of invention data. Patent information is widely used to assess technology development, forecasting, and decision-making.

The research information base was an array of patent documents issued for the period of 2000–2023 (as of 31 August 2023), presented in the Lens database. To analyze patents reflecting the main trends in technical solutions in the field of pulse oximeters, a search was conducted using the following keywords “oximeter” and “oxygen saturation”, using the OR operator. The request was limited to the sections of the Cooperative Patent Classification.

**Figure 1:**
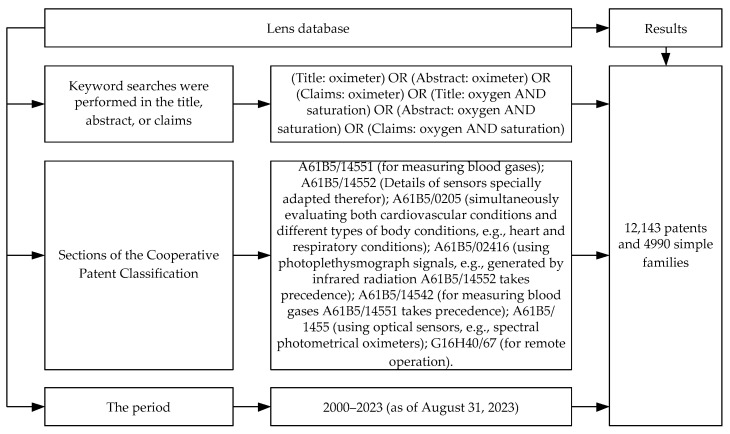
The procedure for determining documents for patent analysis.

Keyword searches were performed in the title, abstract, or claims. The procedure for determining documents for patent analysis is presented in Figure 1.

### 2.2. Bibliometric Analysis

**Figure 2:**
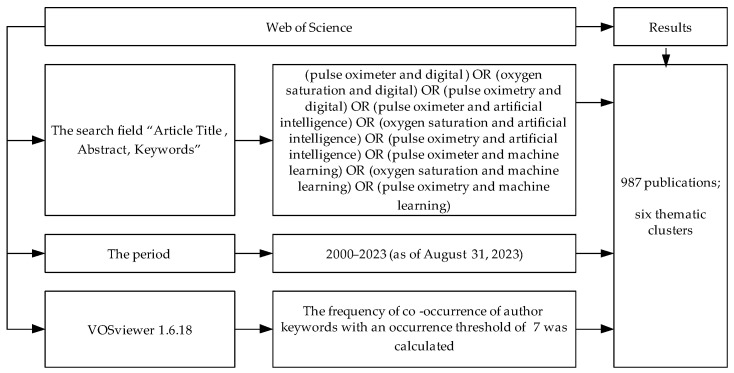
The procedure for defining documents for bibliometric analysis.

For the bibliometric analysis of scientific publications on pulse oximetry, the Web of Science database was used. The period of 2000–2023 (as of 31 August 2023) was also selected as the time interval for the analysis. In order to assess the prospects for using digital technologies in combination with pulse oximeters, the keywords “pulse oximeter”, “oxygen saturation”, “pulse oximetry”, “digital”, “artificial intelligence”, and “machine learning” were used to search. The search field “Article Title, Abstract, Keywords” was used. The procedure for defining documents for bibliometric analysis is shown in Figure 2.

VOSviewer 1.6.18 [31] was used to analyze and visualize the results. The frequency of co-occurrence of author keywords with an occurrence threshold of 7 was calculated. VOSviewer allows one to generate bibliometric maps based on the analysis of the frequency of co-occurrences of keywords, co-citations, and other parameters. In particular, using VOSviewer, conceptual maps were created based on the frequency of co-occurrence of each pair of terms. The size of the circle reflects the frequency of occurrence of the term; the larger its area, the more often this word or phrase is found in the general list of author keywords. The distance between a pair of terms is an indication of their relationship. Colors are used to refer to clusters; terms of the same color (included in the same cluster) are more common with each other (i.e., more closely related) than terms labeled with other colors.

## 3. Results

### 3.1. Patent Analysis

**Figure 3:**
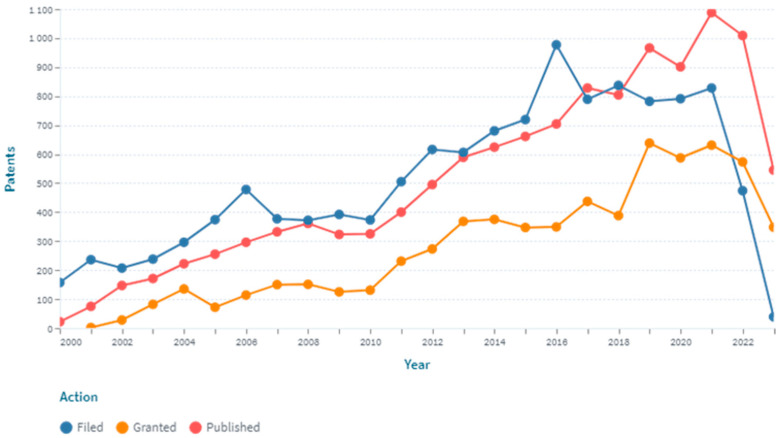
Dynamics of patent activity in the field of creating pulse oximeters for the period of 2000–2023 (as of 31 August 2023).

Patent analysis using a search query of the study revealed 12,143 patents and 4990 simple families for the period from 2000 to 2023 (as of 31 August 2023). The dynamics of patenting inventions in the field of creating pulse oximeters in 2000–2023 are shown in Figure 3.

The presented data indicate the presence of a stable linear growth of patent activity in the field of pulse oximetry, which is probably due to the constant demand for pulse oximeters and the search for new solutions for their improvement. Noteworthy is the sharp increase in the number of published patents in 2021, which is possibly due to the widespread use of pulse oximeters during the COVID-19 pandemic for early detection of hypoxia, both by health personnel and patients at home.

**Figure 4:**
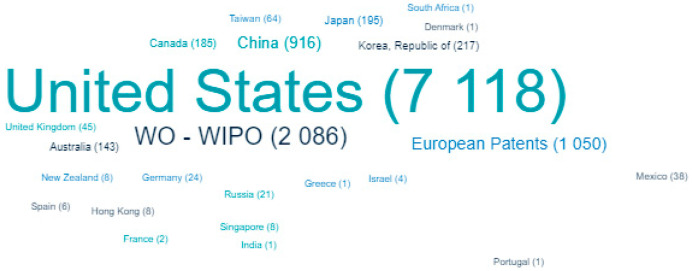
Leading countries by number of patents issued (2020–2023, as of 31 August 2023).

Figure 4 shows the patenting activity in the field of creating of pulse oximeters in various countries. An analysis of the Lens patent database showed that the leading countries issuing patents on their territory were the United States, China, the Republic of Korea, Japan, Canada, Australia, Taiwan, and the United Kingdom. Applicants also made extensive use of the Patent Cooperation Treaty and a procedure for obtaining European patents.

**Figure 5:**
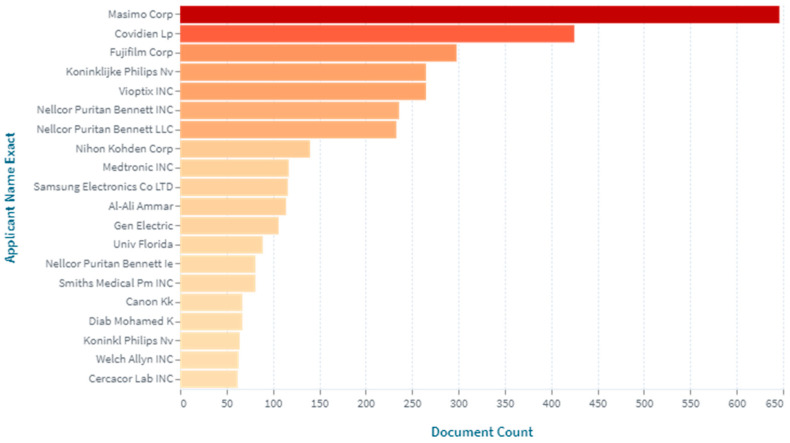
The top applicants in the field of creating pulse oximeters (2000–2023, as of 31 August 2023).

The leading applicants in the field of pulse oximeters were evaluated. It was revealed that the top applicants in this field were Masimo Corporation, Covidien Lp, Fujifilm Corporation, Koninklijke Philips Nv, Vioptix Inc., Nellcor Puritan Bennett, Nihon Kohden Corporation, Medtronic Inc., etc. (Figure 5).

As shown by the patent analysis of innovative solutions for creating pulse oximeters, Masimo is one of the leading development companies. Masimo is an American company founded in 1989 that manufactures devices for non-invasive patient monitoring, including pulse oximeters. Based on earnings growth, revenue growth, and return on equity, Forbes included Masimo in its list of the 20 biggest billion-dollar public corporations in 2011. Joe Kiani, founder of the company, was honored with the Ernst and Young National Entrepreneur of the Year Award and the Life Sciences Award in 2012. In the development and marketing of non-invasive patient-monitoring devices, Kiani was recognized for “revolutionizing the healthcare business” [32]. Masimo received patents for pulse oximeters for different end consumers.

Using the example of a number of leading developers, we will consider the directions for creating technical solutions in the field of pulse oximeters.

The analysis revealed multiple classifications of pulse oximeters. Transmission and reflectance pulse oximeters are currently used in clinical practice. Their work is based on the ability of blood hemoglobin to absorb light emitted from the LED of various wavelengths, which is scattered, reflected by tissues and blood, and reaches the photodetector.

Depending on the end-user, there are a number of pulse oximeters (stationary, portable, for home use, pediatric pulse oximeters, etc.), each of which has its own purpose and features of use.

In a stationary version, pulse oximeters are often integrated into more complex devices (patient monitors, ventilators). Devices of this class are complex systems with extensive functionality. Most of the patient’s monitors are equipped with a large graphic screen, which displays current information about the patient’s condition: blood pressure, a cardiogram in real time, saturation value, and a photopletismogram of blood flow in the body site to which the sensor is attached. Stationary pulse oximeters are used exclusively in medical institutions and work from the electrical grid. These are the largest of all pulse oximeters, but they typically provide the highest accuracy. They are used during operations, in intensive care, and during critical procedures.

For example, Masimo has developed and implemented the Root with O3 Regional Oxymetry Stationary Device for use in adults, children, infants, and newborns for cerebral and somatic monitoring. The patent protection of the device is comprehensive, including protection of the following inventions: “Dual-mode pulse oximeter” (US6770028 [33]); “Low power pulse oximeter” (US7295866 [34]), “Pulse oximeter probe-off detector” (US7471969 [35]), “System and method for monitoring the life of a physiological sensor” (US7880626 [36]), “Robust alarm system” (US7962188 [37]), “Virtual display” (US7990382 [38]), “Plethysmograph pulse recognition processor” (US7988637 [39]), “Pulse oximetry data confidence indicator” (US8046040 [40]), and others [41,42,43,44,45,46,47,48,49,50]. The Root with O3 Regional Oximetry device can help doctors monitor brain oxygenation.

Portable pulse oximeters are used to evaluate oxygen saturation in the treatment of cardiovascular problems both in the hospital and at home. A portable pulse oximeter is placed on the finger, and the sensor is attached to a monitor mounted on the belt. With such a device, it is possible to obtain accurate measurement results during the day, continuously monitoring the patient’s state. Continuous monitoring of saturation can be a key component in preparing for surgery or selecting the right treatment.

An example of a portable pulse oximeter is the iSpO2, developed by Masimo. When measured with an iSpO2 pulse oximeter, the accuracy of monitoring oxygen saturation and the pulse rate is guaranteed even during movement. It works with either iOS or Android devices. The Masimo Personal Health app, combined with the iSpO2 pulse oximeter, allows patients to monitor and change oxygen saturation, pulse rate, and perfusion index. The iSpO2 pulse oximeter is also protected by several patents [34,51,52,53,54,55,56].

A separate niche can be identified for pulse oximeters, focused on a quick assessment of the patient’s condition rather than long-term monitoring. These devices have several features: their miniature size, limited functionality, and ease of use. Such pulse oximeters can be made with a sensor in the form of a clip, which is usually fixed on the index finger or earlobe of the patient. This type is well-suited for adults and adolescents when the patient is observed for a short time. It is inconvenient to wear a clip if one needs a long measurement (several hours or more), as it can shift during movements, distorting the results of the study. For use, it is necessary to be at rest and not move, which makes it impossible to use this type to obtain an average oxygen saturation value for some time.

As a leading development company, Masimo also developed the MightySat finger pulse oximeter to quickly assess a patient’s condition, measuring five key vital signs: oxygen saturation, pulse rate, respiratory rate, perfusion index, and plethistic variability index. These settings are displayed on compatible smart devices with Bluetooth LE models. Measurements can also be integrated into the Apple Health app. This development is protected by several patents ([57,58,59,60,61], etc.).

Pulse oximeters for rapid state assessment are also presented in the form of a wristwatch with a remote sensor that is attached to the finger, as in previous types. Such devices are more often used by athletes and people involved in climbing mountains.

Noteworthy is the development of the Masimo W1 (Advanced Health Tracking Watch). Numerous physiological measurements are provided by Masimo W1, including the hydration index, oxygen saturation, heart rate, respiration rate, pleth variability index, and perfusion index. The product is made for people who wish to track their health data on their own or with friends and family, make better fitness decisions, or follow their health information with more knowledge. The watch is protected by a few patents [62,63,64,65].

The company’s products utilize Masimo SET technology, which should be highlighted. With the use of cutting-edge signal processing methods, SET sets Masimo apart from existing pulse oximetry systems by separating the arterial signal from noise sources. Masimo SET, when used in conjunction with clinical controls, has been shown in numerous studies to assist doctors in lowering the risk of blindness and eye damage (retinopathy of prematurity) in newborns [66], improving newborn critical congenital heart disease screening [67], and lowering the number of patient transfers to intensive care units and the activation of a rapid response team on the general hospital floor [68].

In a patient’s physical data file, health care providers often record numerical parameter values together with other crucial data, such as time, other observational data, or remarks, and the identity of a health care professional. The file is frequently kept close by the patient, perhaps on a tablet. In this instance, the file is accessible to individuals who want to view the data. It should be mentioned that computer pulse oximetry is used in relation to this, where data processing from the device occurs through a microprocessor embedded into the device. This design is seen in many contemporary pulse oximeters.

Masimo is actively working towards the introduction of digital pulse oximetry technologies. A remote monitoring and notification system for doctors (Masimo Patient SafetyNet) has been developed, which displays information in almost real-time from any connected Masimo device or a third-party device at the central station. The system allows for the sending of alarms and alerts from bedside devices directly to doctors. The development is protected by a number of patents, including “Intelligent medical escalation process” (US10833983 [69]), “Physiological measurement logic engine” (US11399774 [70]), “Medical communication protocol translator” (US11145408 [71]), “Medical monitoring system” (US11133105 [72]), and “Alarm notification system” (US11488711 [73]).

Moreover, Masimo’s Iris Gateway system bridges the gap between device data generated at the patient’s bedside and documentation in patient data-management systems such as electronic EMR medical records. Masimo Iris contributes to simplifying interaction throughout the health care delivery process. The development is presented in a number of patents, including “Physiological measurement communications adapter” (US7844315 [74]), “Systems and methods for storing, analyzing, and retrieving medical data” (US8274360 [75]), “System for displaying medical monitoring data” (US9943269 [76]), etc.

Furthermore, Masimo has presented an original, innovative solution that uses pulse oximetry that is noteworthy and of great practical importance. This is an opioid overdose prevention and warning system disclosed in patent US10939878 [77]. Using a pulse oximeter at the tip of the finger attached to an intelligent mobile device, the physiological monitoring system tracks breathing based on oxygen saturation measurements and communicates opioid-monitoring data from an intelligent mobile device to an opioid overdose monitoring service.

Masimo also develops pulse oximeters for sports events. An application (US 2022/0218244 A1) has been made for the invention, which relates to methods and systems for combining physiological data from a pulse oximeter connected to a player playing a tennis match with match data corresponding to a tennis match [78]. The system can generate a visual alert for one or more spectators of a tennis match based on a trigger event corresponding to the received physiological data and match data.

It should be noted that the identified areas of development in the field of pulse oximeters for different groups of end consumers are also presented among the intellectual property portfolios of other leading companies.

The founders of the creation of pulse oximeters include Nellcor, created in 1981. In 1983, Nellcor introduced its first pulse oximeter. As a result of mergers and acquisitions involving Nellcor, the following companies were represented in the market: Puritan-Bennett, Nellcor Puritan Bennett, and Covidien. In 2015, Medtronic acquired Covidien and continues to promote the Nellcor brand [79]. As a result, the intellectual property market is widely represented by the above applicants.

The Nellcor pulse oximeter that has proven itself positively on the market is the Oxi Max N 560. It is designed to continuously and non-invasively monitor the degree of functional oxygen saturation of the arterial hemoglobin and the pulse rate. The device N-560 is intended for newborns, children, and adults both in and out of motion, as well as for patients in well-lit or poorly-lit rooms, such as hospitals, health facilities, in-hospital transport, and home settings. The Oxi Max N 560 is protected by a number of patents, including “Sensor with signature of data relating to sensor” (US6708049 [80]), “Method and circuit for storing and providing historical physiological data” (US6463310 [81]), “Method and circuit for indicating quality and accuracy of physiological measurements” (US6675031 [82]), “Pulse oximeter sensor with piece-wise function” (US6801797 [83]), “Oximeter sensor with digital memory recording sensor data” (US6591123 [84]), etc.

Of particular interest is the development of Covidien Lp, protected by the patent “Oxygen Saturation Monitoring Using Artificial Intelligence” (US11517226) [85]. The developed oxygen saturation-monitoring device determines that the oxygen saturation level of the patient has decreased before reaching the desaturation threshold and can predict whether the oxygen saturation level of the patient will rise again above the desaturation threshold within a predetermined period.

In the field of pediatrics, Covidien Lp has received a patent for a device for determining the risk of retinopathy of prematurity (US8374666) [86]. The processing unit is configured to determine the oxygen saturation level and trigger an alarm to receive timely information about the emerging risk.

Covidien Lp also applied for WO 2022/204668, “Autoregulation Monitoring Using Deep Learning” [87]. In anesthesia, that is particularly interesting in patients that are positioned with their heads elevated during surgery. The system is configured to determine, using a neural network algorithm, a model of cerebral autoregulation. A patient’s cerebral autoregulation state is based at least in part on the patient’s blood pressure and regional cerebral oxygen saturation in the patient for a certain period of time.

The leaders among the developers of pulse oximeters include Nihon Kohden. In 1972, the Japanese engineer Takuo Aoyagi developed a method for recording fluctuations in the absorption of light during the pulsation of the arteries. In 2015, he was awarded by the Institute of Electrical and Electronics Engineers with the “Healthcare Technology Innovation Medal,” being the first Japanese to receive such an award. The first pulse oximeter was released in 1975 (model OLV-5100) by Nihon Kohden Corporation [88]. Nihon Kohden currently has a large portfolio of pulse oximeter patents, including “Pulse oximeter” (US 7206621 [89]), “Method and Apparatus for Measuring Pulse Rate and Oxygen Saturation Achieved During Exercise” (US 8649837 [90]), “Patient Monitor” (US 11291414 [91]), etc.

### 3.2. Bibliometric Analysis

**Figure 6:**
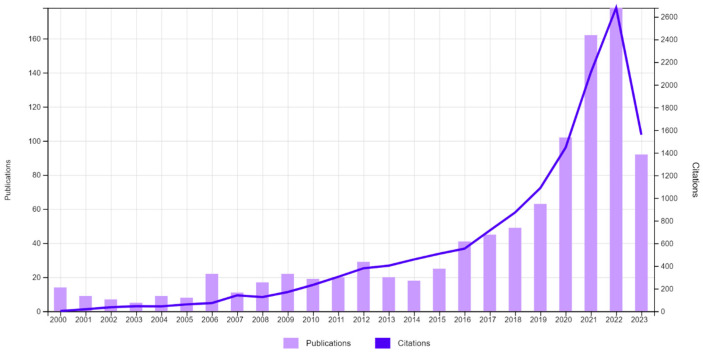
Dynamics of the number of publications and citations devoted to pulse oximetry using digital technologies in the Web of Science (2000–2023, as of 31 August 2023).

Based on the entered search queries in the Web of Science database, the system provided 987 publications. Bibliometric analysis, as shown in Figure 6, revealed a time-stable growth trend in the number of publications and citations devoted to pulse oximetry using digital technologies.

Figure 6 shows that 54% of all materials have been published since 2020. A total of 54% of scientific publications on pulse oximetry using digital technologies cover the following areas of knowledge: electrical engineering (13.982%), biomedical engineering (12.563%), general internal medicine (8.004%), medical informatics (7.497%), health care sciences services (6.180%), and instruments instrumentation (5.978%). Among the medical industries, leading positions occupy respiratory system (4.154%), surgery (3.951%), cardiac cardiovascular systems (3.850%), anesthesiology (3.749%), critical care medicine (3.546%), and clinical neurology (3.141%).

Among these 987 papers, 718 were articles, 67 were reviews, 195 were proceeding papers, and 6 were editorial materials.

The top five most productive affiliations and journals are listed in Table 1.

It was determined that the USA, China, England, India, Germany, Japan, Canada, Australia, Spain, and Italy are the countries in which the problems of pulse oximetry were worked out in the most detail. Three countries (the USA, China, and England) produced almost half of the total number of publications (50.8%).

The five most cited publications of authors studying pulse oximetry in combination with digital technologies in the Web of Science (2000–2023, as of 31 August 2023) are shown in Table 2.

The most cited (678 citations) is the publication “Ultraflexible organic photonic skin” by Yokota et al. [92]. In this study, the authors reviewed the development of thin-film electronic devices for health monitoring. An ultraflexible reflex pulse oximeter was made.

In second place is the publication “Photoplethysmography: Beyond the Calculation of Arterial Oxygen Saturation and Heart Rate” [93]. It has been quoted 300 times. This study carried out a detailed analysis of photoplethysmography. In addition, the need to standardize and quantify the plethysmograph, device improvements, and well-designed prospective studies demonstrating the measurement of clinically-relevant information were emphasized.

In third place is the publication “Prediction of Sepsis in the Intensive Care Unit with Minimal Electronic Health Record Data: A Machine Learning Approach” by Desautels et al. [94], which has been cited 226 times. The paper proposed a method for predicting sepsis in intensive care using machine learning based on a minimal set of variables from electronic medical record data (including pulse oximetry data). The next highly cited publication [95] relates to the use of photoplethysmograms in blood circulation monitoring for new technological developments.

A highly cited study by Li et al. demonstrates that with portable devices, diverse measurements can be systematically obtained and used to monitor health-related physiology and activity [96]. These measurements are likely to be important not only in basic scientific research but also in clinical settings. It is likely that in the future, these devices will be used by physicians to assess health conditions and make recommendations and treatments.

The top 20 keywords by frequency of occurrence are presented in Table 3.

**Figure 7:**
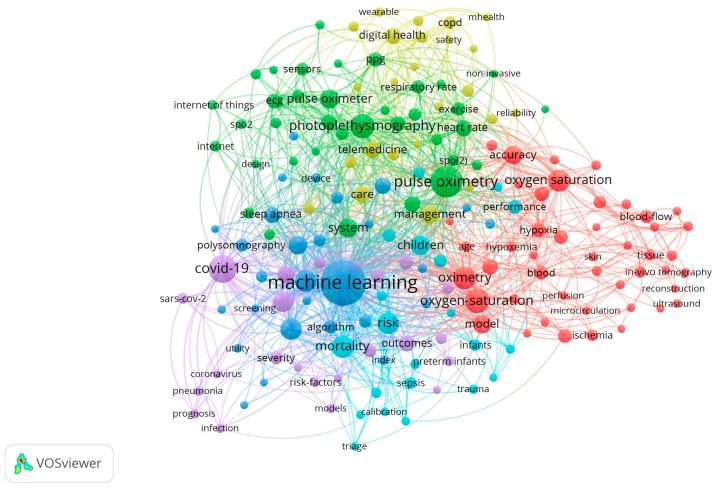
A conceptual map of the keywords of publications on pulse oximetry using digital technologies in Web of Science (2000–2023, as of 31 August 2023).

Using the method of clustering the keywords of the VOSviewer program, a conceptual map was created (Figure 7). It shows that terms form a complex network in which six thematic clusters can be distinguished.

## 4. Discussion

The final stage in the development of devices, including innovative technologies for pulse oximeters, is their testing and introduction into medical practice. Thus, for example, Masimo Rad-97 and Accessories, Masimo Radical-7 Pulse CO-Oximeter and Accessories, Masimo Radius-7 Pulse CO-Oximeter and Accessories, Masimo Rad-67 Pulse CO-Oximeter and Accessories, and others have received FDA approval (https://www.accessdata.fda.gov/scripts/cdrh/cfdocs/cfRL/rl.cfm (accessed on 24 September 2023)).

There must be a focus on the FDA guidelines, which determine the following types of pulse oximeters [26]:

- Pulse oximeters are used by prescription and have passed clinical trials. They are used most often in hospitals and doctors’ offices, and less often for home use.
- Over-the-counter pulse oximeters. They do not pass FDA review and are more often intended for general wellness, sports, or aviation purposes.

Analysis of the results of patent studies revealed that modern innovative solutions for the creation and implementation of transmission and refractory pulse oximeters for different end-users (stationary, portable, for home use, for pediatrics, etc.) are often integrated into more complex devices. At the same time, the tendency to accompany them with digital technologies is clearly traced. Manufacturers emphasize the development of automated, wireless, and remote pulse oximeters, thereby reducing the number of hospitalizations and doctor’s visits and improving diagnosis and treatment.

In order to analyze and systematize the available data on the experience of introducing pulse oximeters using digital technologies, bibliometric studies were carried out.

Ongoing studies reveal new beneficial effects of pulse oximetry in clinical practice. The bibliometric analysis of pulse oximetry revealed six areas of research, as well as technical solutions to reduce the risks associated with pulse oximetry.

The first cluster (marked in red) is associated with the accuracy of the measurement results obtained by pulse oximeters. The predominant terms include accuracy, age, blood flow, hemodynamics, circulation, hemoglobin, hypotension, hypoxemia, hypoxia, near-infrared spectroscopy, perfusion, reflectance, skin, temperature, etc.

Cabanas et al. systematized factors that may influence the accuracy and interpretation of pulse oximeter readings. Conditions of low perfusion, such as hypotension, hypothermia and vasoconstriction, anemia, hypoxemia, the use of dyes, nail polish, nerve-blocking medications, high altitude, or hypoxia training, can lead to SpO2 underestimation [97].

Although some older studies have not found any abnormalities associated with dark skin pigmentation [98,99], recent studies have shown that pulse oximeter devices have some limitations for dark-skinned subjects, especially in low-saturation or hypoxemia settings. Cabanas et al. note that factors that can lead to overestimation are darker skin pigmentation, hot skin temperature, hemoglobinopathy, high carbon monoxide levels, hyperbilirubinemia, and heavy smokers [23,97].

Additionally, the pulse oximeter’s signal processing can be hampered by motion artifacts, HR >150 beats per minute, excessive ambient light, incorrect or loose placement of the probe on the finger or earlobe, electromagnetic interference, or irregular heart rhythms, all of which can lead to readings that are not accurate [97,100].

The FDA notes that some of the factors that may affect the accuracy of pulse oximeters include skin pigmentation, dyshemoglobinemia, severe anemia, low perfusion, dyes, nail polish, ambient light, motion artifacts, etc. [26].

In publications containing the above keywords, problems with the measurement accuracy of pulse oximeters and the usefulness of digital support are discussed. For example, Gokhale et al. have developed a non-invasive technology for measuring hemoglobin and oxygen saturation to eliminate racial bias [101]. The inventors use a special melanin-accounting algorithm and software. Non-invasive device scores were comparable to the invasive method.

The work of Matos et al. was also devoted to the study of the accuracy of pulse oximeters in a population with a darker skin tone [102]. The results of their single-center study suggest that SpO2 correction can be achieved with machine learning.

Other authors report that in neonatal intensive care units, 87.5% of monitoring system alarms are false and caused by neonatal movements. To reduce the level of false alarms, it is proposed to use machine-learning algorithms to analyze the data from standard physiological monitoring in combination with cerebral oximetry data [103].

The proposed digital technologies using machine learning might help solve the problems of the influence of pigmentation and motion artifacts on measurement accuracy.

There are other developments in pulse oximeters that use more wavelengths, which increase their accuracy. In the future, machine learning methods can be applied to these data. Thus, Rea et al. noted that the inclusion of various specific wavelengths allows the reliable use of noninvasive sensors by people of any race and/or skin type [104].

The second cluster (green) focuses on the capabilities of the Internet of Medical Things, including pulse oximeters. Biomedical monitoring, exercise, heart rate, Internet of Things, monitoring, motion, respiratory rate, sensor, SpO2, vital signs, wearable devices, and wearable sensors are the terms that are presented in this cluster.

Biosensors, as a general category of devices, collect information about different parameters and vital health signs by reading or measuring them and transmitting them using electrical signals to be interpreted. Pulse oximeters are of interest for the prevention and control of diseases, including those in elderly patients. They provide monitoring outside the hospital. At the same time, the indicators are more objective than those reported by patients during their visits to the doctor and can provide specialists with a real picture of the course of the disease in each specific case. The ability to connect sensors to mobile devices allows one to improve health management. It should also be noted that fitness trackers and smartwatches often include pulse oximetry indicators.

It is of interest to use sensors with digital technologies to detect early signs of drug overdose and respond in a timely manner. A review by Oteo et al. focused on technologies that can alert physicians to cases of drug overdose [105]. These technologies use sensors that monitor oxygen saturation levels, breathing rate, or movement in combination with smartphone applications.

Scientific evidence suggests that one in five Americans wears a fitness tracker [106]. The publications of this cluster are also devoted to the development and testing of smartwatches, including readings of pulse oximeters.

The main users of smart watches are professional athletes and sports fans, military personnel, and users monitoring health. Thus, Garmin Ltd. produces smartwatches for sports and the military sphere [107].

Mitro et al. developed and tested a smart bracelet with machine learning support for use in emergencies during the evacuation of large passenger ships [108]. The device allows one to monitor the pulse rate and oxygen saturation level in real-time and detect stress. An external review established an accuracy score of 76%.

Studies on the accuracy of oxygen saturation measurements using the Apple Watch (Apple Inc., Cupertino, CA, USA) were summarized [109]. A total of 973 patients had a 95% agreement of ±2.7 to 5.9% SpO2 when using the Apple Watch Series 6. The limitations of the studies were heterogeneous measurement and reporting processes in comparison with non-invasive measurements at rest. The authors noted the need to study the effect of skin color on measurement accuracy. In the clinical setting, a 12-lead ECG is currently needed to diagnose a myocardial infarction (MI). Li et al. summarized data from a number of studies using the Apple Watch to record multiple leads to meet this requirement for a clinical diagnosis of MI. The researchers noted that there are still many limitations to achieving the goal of early detection of MI, and more clinical data are needed [110].

It is worth noting that the Apple Watch and other wrist devices are not class IIb medical devices. While class IIb devices are generally medium- to high-risk, this class is more complex due to stricter clinical data requirements [111,112].

The determination of oxygen saturation by these wrist devices is only punctual and not continuous, and the patient/user must remain still.

When using smart watches for medical purposes, scientists distinguish between the positive aspects of the high-tech functions of smart watches, which allow users to independently monitor their health, and the risks associated with the error of medical indicators recorded by smart watches. There is no doubt that the potential of smart devices will increase, and their data will be comparable to the results of stationary examinations.

The third cluster (blue) is associated with the study of the use of pulse oximeters with digital technologies in various pathologies. This cluster has the following terms: obstructive sleep apnea, diabetes, diagnosis, digital signal processing, machine learning, neural networks, polysomnoraphy, predictiob, screening, stroke, support vector machine, etc. In recent years, pulse oximetry using digital technologies has become actively used as a diagnostic tool in clinical practice and in functional diagnostics.

Pulse oximetry using digital technologies can be used as a screening method to determine sleep apnea. A systematic search by Bazoukis et al. revealed that machine-learning models performed well in the diagnosis of sleep apnea using electrocardiogram measures and pulse oximetry [113].

Wang et al. developed an apnea diagnostic system using a photopletismography sensor to synchronously collect human pulse wave signals and oxygenate the blood. Machine learning was used to process data [114]. The high accuracy of the system was revealed—more than 85%.

Pulse oximetry using digital technologies in combination with other indicators is a fast and simple, but to the same extent informative, method of studying the function of the cardiovascular system. Its use is possible both in emergency cases (operating room, ambulance) and in the diagnosis of chronic diseases. In the initial stages of the disease, in some cases, only hypoxemia may indicate a developing pathology.

Chu et al. proposed a transformer-based deep-learning architecture that uses photopletysmogram signals to conduct personalized blood pressure assessments and oxygen saturation [115]. The model meets clinical standards and can improve the accuracy of blood pressure and oxygen saturation measurements (n = 1732).

Huang et al. presented a machine-learning model predicting 28-day all-cause mortality in hypertensive ischemic or hemorrhagic stroke patients [116]. Indicators included age, ethnicity, white blood cells, hyperlipidemia, mean corpuscular volume, glucose level, oxygen saturation, serum calcium, red blood cell distribution width, blood urea nitrogen, and bicarbonate (n = 4274).

Kerexeta et al. proposed an artificial intelligence model predicting the risk of cardiac decompensation events [117]. The prognostic indicators selected were weight gain, oxygen saturation in the last few days, and heart rate.

Pulse oximetry using digital technologies has found application for detecting hemodynamically significant disease of peripheral arteries of the lower extremities in patients aged 50 years and older with type 2 diabetes [118].

The use of pulse oximetry with digital technologies in various areas of medical practice improves the work of identifying episodes of reduced oxygenation and thereby significantly increases the timeliness and quality of diagnosis of threatened conditions and the quality of medical care. Machine learning methods are increasingly being used to mine data characterizing the clinical status of patients.

The central topics of the fourth cluster (yellow-green color) are telemedicine and mobile applications to ensure the safety of patients in the provision of medical care, including using pulse oximeters. In this cluster, the keywords are asthma, COPD (chronic obstructive pulmonary disease), digital heath, management, Mhealth, mobile health, patient monitoring, physical activity, prevention, reliability, remote monitoring, safety, telehealth, telemedicine, therapy, validation, and wearable.

Telemedicine and remote monitoring contribute to improving the quality and safety of the medical care provided, taking into account continuous modifications and improvements in diagnostic and therapeutic tools, including the growing number of chronic patients. This cluster presents publications related to the above topic.

Ashfaq et al. developed a prototype for the remote monitoring of cardiovascular patients using machine learning and artificial intelligence techniques [119]. The device allows the monitoring of vital human functions, including oxygen saturation, heart rate, and body temperature.

It was reported that the remote monitoring of vital parameters and symptoms (blood pressure, respiratory rate, heart rate, temperature, dyspnea, and peripheral saturation) is safe and allows patients hospitalized for COVID-19 to be discharged from the hospital ahead of schedule [120].

The use of monitoring patients for asthma was analyzed [121]. Electrocardiogram and photoplethysmogram signals are widely used in smartwatches and breast bracelets, making it easy to integrate a more extensive body sensor system to predict asthma exacerbations. Blood oxygen saturation, temperature, blood pressure, verbal sounds, and pain responses are other vital indicators that are utilized to monitor asthma patients.

Digital pulse oximetry indicators are essential for determining the treatment tactics and prognosis for patients with respiratory pathology, particularly those with chronic obstructive pulmonary disease [122].

Telemedicine and mobile applications reduce the burden on medical staff and increase the safety of patients.

The fifth cluster (lilac color) is closely related to the third and focuses on the possibilities of applying artificial intelligence and deep learning to analysis and prediction in COVID-19 and neonatology, including using pulse oximeters. Key terms include: artificial intelligence, complications, coronavirus, COVID-19, deep learning, disease, deep learning, outcomes, pain, pneumonia, prediction model, preterm infants, retinopathy of premature, severity, and SARS-CoV-2.

The COVID-19 pandemic has contributed to a significant increase in interest in the use of digital technologies in general and artificial intelligence in particular in the context of healthcare [123,124,125]. The use of pulse oximetry using digital technologies is due to the fact that arterial hypoxemia is one of the main syndromes that develops in patients with pneumonia caused by COVID-19.

Using various machine learning approaches, the prognostic value for survival of COVID-19 patients based on known in-hospital mortality risk factors and chest radiographs was assessed. It was found that the indicators should include age, oxygen saturation, blood pressure, and some concomitant diseases, as well as image features related to the intensity and variability of the pixel distribution [126].

In connection with the COVID-19 pandemic, patients had to manually enter their daily oxygen saturation and pulse rate values into a health monitoring system. In order to optimize this process, a new PACMAN (pandemic-accelerated human–machine collaboration) structure with low-resource computer vision based on deep learning has been proposed and investigated. The structure is integrated into the patient monitoring system [127].

Considering that patients with COVID-19 may have significantly low SpO2 before obvious symptoms appear, Mathew et al. proposed neural network-based monitoring methods using smartphone cameras [128]. The device analyzes the participant’s hand video for physiological indicators.

Due to the similarity of the symptoms of COVID-19 and other respiratory infections, the diagnosis of these diseases may be difficult. To solve this problem, a web application was developed that uses a chatbot and artificial intelligence to detect COVID-19, colds, and allergic rhinitis. The app also includes an electronic device that connects to the app and measures vital signs such as heart rate, blood oxygen saturation, and body temperature [129].

Pulse oximetry occupies a special place in neonatology. A prognostic algorithm has been created to detect late-onset sepsis in preterm infants [130]. The machine-learning model takes into account oxygen saturation and heart rate data with a per-minute sampling rate.

In order to reduce the development of retinopathy, the importance of using improved saturation monitors in the management of newborns in critical conditions is noted. The introduction of automated systems is promising, helping to regulate the oxygen blender based on digital oxymetry data in real-time [131].

Thus, pulse oximetry with digital technologies is highly specific for detecting critical conditions in COVID-19 and neonatology.

The sixth cluster (light blue) concerns the experience of using pulse oximeters with digital technologies in the departments of anesthesiology, resuscitation, and intensive care. Among the keywords, the following concepts can be distinguished: anesthesia, calibration, children, critical care, failure, in-hospital mortality, newborn, risk, sepsis, shock, support, surgery, and trauma.

To optimize the processes of diagnosis and treatment in intensive care, machine learning, accompanied by various devices, including pulse oximeters, can also be successfully used. In order to reduce the duration of intraoperative hypoxemia in pediatric patients, a machine-learning model has been developed and tested that could predict the events of intraoperative hypoxemia 1 min ahead in children undergoing general anesthesia [132].

A machine-learning model has been developed and tested that predicts short-term ICU mortality using trends in four easily collected vital signs [133]. Heart rate, systolic blood pressure, diastolic blood pressure, and peripheral capillary oxygen saturation datasets measured every hour for 10 h were used.

Hypoxemia often occurs in outpatients undergoing esophagogastroduodenoscopy under anesthesia. To predict the risk of hypoxemia, Fang et al. developed and evaluated four machine-learning models based on preoperative and intraoperative data. The models showed satisfactory prognostic characteristics [134].

A model for predicting mortality in critically-ill patients with cardiogenic shock (n = 8815) was developed using a machine learning method. The final risk assessment includes the following values during the first 24 h of intensive care unit stay: maximum blood urea nitrogen ≥ 25 mg/dL, minimum oxygen saturation <88%, minimum systolic blood pressure < 80 mmHg, use of mechanical ventilation, age ≥ 60 years, and maximum anion difference ≥14 mmol/L [135].

One of the problems in the departments of anesthesiology, resuscitation, and intensive care is sepsis, since its development not only worsens the course of the patient’s underlying disease but also has a high overall mortality (approx. 30%). The development of machine learning technology and the use of big data in the departments of anesthesiology, resuscitation, and intensive care have prospects for solving this problem.

Thus, in the work of Strickler et al., statistically significant data on the prognosis of the development of sepsis were obtained [136]. A mechanism for interpreting machine-learning models using 17 features was proposed. The authors noted that age, chloride ion concentration, pH, and oxygen saturation should be further investigated for associations with the development of sepsis.

Machine-learning algorithms that analyze data, including pulse oximeters, in real-time are a means of predicting the development of adverse incidents in intensive care in advance, creating the possibility of adequate correction of the patient’s condition in order to avoid further deterioration.

**Figure 8:**
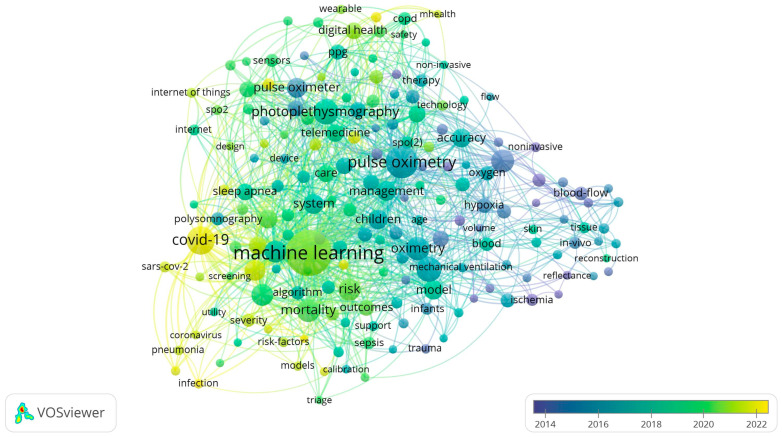
A conceptual map of the keywords of publications on pulse oximetry using digital technologies in the Web of Science in a time aspect.

The VOSviewer program also allows researchers to display the time when the most-common terms in research appear. The closer to blue, the “older” the research (earlier publications); the closer to yellow, the more modern (recent publications). The data obtained are presented in Figure 8.

It should be noted that the time range was automatically reduced by the program due to fewer publications for 2000–2013. This analysis showed that the growth of publishing activity related to machine learning and telemedicine with the use of pulse oximeters actively began in 2020.

**Figure 9:**
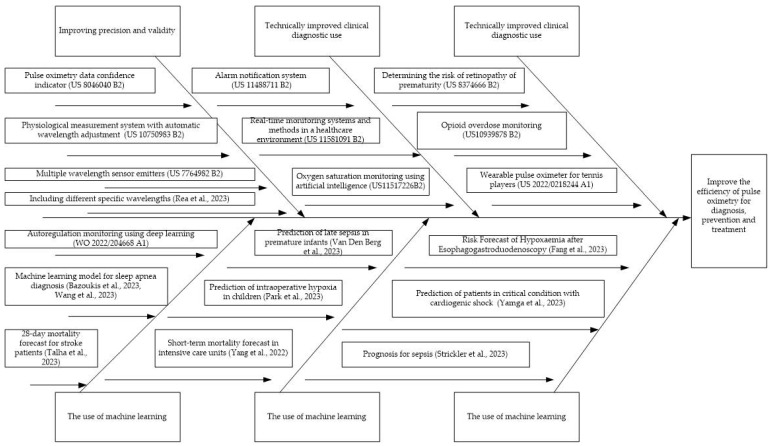
Generalized approaches to risk reduction when using pulse oximetry based on the example of a number of patents and scientific articles [104,113,114,120,130,132,133,134,135,136].

The conducted patent and bibliometric analysis allowed the identification of technical solutions to reduce the risks associated with pulse oximetry: improving precision and validity, technically improved clinical diagnostic use, and the use of machine learning. Generalized approaches to risk reduction when using pulse oximetry on the example of a number of patents and scientific articles are shown in Figure 9.

Thus, the rational choice of strategy to reduce the risks associated with the use of pulse oximetry will allow developers and doctors to support and expand the quality and effectiveness of diagnostic, preventive, and therapeutic practices.

Our patent and bibliometric analysis of the scientific and intellectual property landscape of the use of pulse oximeters indicates their active development and implementation in medical practice. Currently, the large array of pulse oximeters can be classified according their application—from diagnostic to preventive (e.g., for a healthy lifestyle, a smart watch), and a trend in rehabilitation devices, such as monitoring of opioid overdoses.

The integration of digital technologies into pulse oximetry significantly enhances healthcare delivery by streamlining the flow of patient data, improving patient safety, enabling timely medical care, and augmenting the objectivity of clinical results and the accuracy of clinical outcomes, while reducing both the time and use of material resources.

## 5. Conclusions

(1) Patent analysis based on the Lens database for the period of 2000–2023 indicates the presence of a stable linear growth of patent activity in the field of pulse oximetry, with a sharp increase in the number of published patents in 2021, which may be due to the widespread use of pulse oximeters during the COVID-19 pandemic for early detection of hypoxia.

It was revealed that the United States, China, the Republic of Korea, Japan, Canada, Australia, Taiwan, and the United Kingdom are the predominant countries in patent issuance for pulse oximeter technology. Leading companies include Masimo Corporation (Irvine, CA, USA), Covidien Lp (Dublin, Ireland), Fujifilm Corporation (Tokio, Japan), Koninklijke Philips Nv (Amsterdam, Netherlands), Vioptix Inc. (Newark, NJ, USA), Nellcor Puritan Bennett (Carlsbad, CA, USA), Nihon Kohden Corporation (Tokio, Japan), Medtronic Inc. (Dublin, Ireland), etc. A number of their developments have received FDA approval.

It has been revealed that modern innovative solutions for creating and introducing pulse oximeters for various end-users (stationary, portable, for home use, pediatrics, etc.) are currently being integrated into more complex devices with a clearly traceable trend of accompanying them with digital technologies, which makes it possible to reduce the number of hospitalizations and visits to a doctor, as well as improve diagnosis and treatment.

(2) Bibliometric analysis in the Web of Science database for the period of 2000–2023 revealed a consistent temporal trend in the volume of both publications and citations, underscoring the growing importance of pulse oximeters in digitally-enabled medical practice.

The University of California system, Harvard University, the University of Oxford, the Massachusetts Institute of Technology, and the Massachusetts General Hospital were the most productive affiliations. The USA, China, England, India, Germany, Japan, Canada, Australia, Spain, and Italy were the most productive countries. Three countries (the USA, China, and England) produced almost half of the total number of publications (50.8%). The journal “Sensors” was the most productive journal. The most cited literature focuses on measurement accuracy and machine learning.

Using the VOSviewer software, we discerned six primary research clusters: (1) measurement accuracy; (2) integration with the Internet of Things; (3) applicability across diverse pathologies; (4) telemedicine and mobile applications; (5) artificial intelligence and deep learning; and (6) utilization in anesthesiology, resuscitation, and intensive care departments. The findings of this study indicate the prospects for leveraging digital technologies in the use of pulse oximetry in various fields of medicine, with implications for advancing understanding, diagnosis, prevention, and treatment, including intensive therapy. It enables clinicians to proactively identify high-risk patients, including premature infants, who may require more focused diagnostic and therapeutic interventions.

(3) The conducted patent and bibliometric analysis allowed the identification of technical solutions to reduce the risks associated with pulse oximetry: improving precision and validity, technically improved clinical diagnostic use, and the use of machine learning.

In summary, the future of digitally-augmented pulse oximeters is promising. These advances not only have great development prospects, but also contribute to enhancing patient safety and the overall quality of medical care.

## Figures and Tables

**Table 1:** Top 5 most productive affiliations and journals.

Entity	Record Count	%
***Affiliations***		
University of California system	35	3.57
Harvard University	34	3.47
University of Oxford	22	2.25
Massachusetts Institute of Technology	18	1.84
Massachusetts General Hospital	17	1.74
***Journals***		
Sensors	28	2.86
Proceedings of SPIE	23	2.35
PLOS One	18	1.84
Scientific reports	17	1.74
Physiological measurement	13	1.33

**Table 2:** The top 5 most cited publications by authors studying pulse oximetry in combination with digital technologies in the Web of Science (2000–2023, as of 31 August 2023).

Title	Year of Publication	Average per Year	Total Citations
Ultraflexible organic photonic skin	2016	84.75	678
Photoplethysmography: beyond the calculation of arterial oxygen saturation and heart rate	2007	17.65	300
Prediction of sepsis in the intensive care unit with minimal electronic health record data: a machine learning approach	2016	28.25	226
Utility of the photoplethysmogram in circulatory monitoring	2008	14.13	226
Digital Health: tracking physiomes and activity using wearable biosensors reveals useful health-related information	2017	30.29	212

**Table 3:** The top 20 keywords of publications in the field of pulse oximetry using digital technologies in the Web of Science (2000–2023, as of 31 August 2023).

Keywords	Occurrences
Machine learning	200
Oxygen saturation	109
Pulse oximetry	100
COVID-19	76
Photoplethysmography	59
Oximetry	52
Mortality	49
Prediction	46
Risk	46
Diagnosis	45
Artificial intelligence	41
Pulse oximeter	37
Management	35
Accuracy	34
Deep learning	30
Care	28
Telemedicine	28
Digital health	27
Sleep apnea	27
Outcomes	24

## Data Availability

All relevant data are included in the manuscript. Further queries addressed to the corresponding authors are welcome.
